# Improving Rates of Influenza Vaccination Through Electronic Health Record Portal Messages, Interactive Voice Recognition Calls and Patient-Enabled Electronic Health Record Updates: Protocol for a Randomized Controlled Trial

**DOI:** 10.2196/resprot.5478

**Published:** 2016-05-06

**Authors:** Sarah L Cutrona, Meera Sreedhara, Sarah L Goff, Lloyd D Fisher, Peggy Preusse, Madeline Jackson, Devi Sundaresan, Lawrence D Garber, Kathleen M Mazor

**Affiliations:** ^1^ University of Massachusetts School of Medicine Division of General Medicine/Primary Care Worcester, MA United States; ^2^ Meyers Primary Care Institute Worcester, MA United States; ^3^ University of Massachusetts School of Medicine Worcester, MA United States; ^4^ Baystate Medical Center Springfield, MA United States; ^5^ University of Massachusetts Medical School Department of Pediatrics Worcester, MA United States; ^6^ Reliant Medical Group Pediatrics Department Worcester, MA United States; ^7^ Reliant Medical Group Research Department Worcester, MA United States; ^8^ Reliant Medical Group Internal Medicine Department Worcester, MA United States

**Keywords:** electronic health records, influenza vaccines, clinical decision support, Internet, Telephone, Electronic Mail, Health Records, Personal, Medical Informatics Applications

## Abstract

**Background:**

Clinical decision support (CDS), including computerized reminders for providers and patients, can improve health outcomes. CDS promoting influenza vaccination, delivered directly to patients via an electronic health record (EHR) patient portal and interactive voice recognition (IVR) calls, offers an innovative approach to improving patient care.

**Objective:**

To test the effectiveness of an EHR patient portal and IVR outreach to improve rates of influenza vaccination in a large multispecialty group practice in central Massachusetts.

**Methods:**

We describe a nonblinded, randomized controlled trial of EHR patient portal messages and IVR calls designed to promote influenza vaccination. In our preparatory phase, we conducted qualitative interviews with patients, providers, and staff to inform development of EHR portal messages with embedded questionnaires and IVR call scripts. We also provided practice-wide education on influenza vaccines to all physicians and staff members, including information on existing vaccine-specific EHR CDS. Outreach will target adult patients who remain unvaccinated for more than 2 months after the start of the influenza season. Using computer-generated randomization and a factorial design, we will assign 20,000 patients who are active users of electronic patient portals to one of the 4 study arms: (1) receipt of a portal message promoting influenza vaccines and offering online appointment scheduling; (2) receipt of an IVR call with similar content but without appointment facilitation; (3) both (1) and (2); or (4) neither (1) nor (2) (usual care). We will randomize patients without electronic portals (10,000 patients) to (1) receipt of IVR call or (2) usual care. Both portal messages and IVR calls promote influenza vaccine completion. Our primary outcome is percentage of eligible patients with influenza vaccines administered at our group practice during the 2014-15 influenza season. Both outreach methods also solicit patient self-report on influenza vaccinations completed outside the clinic or on barriers to influenza vaccination. Self-reported data from both outreach modes will be uploaded into the EHR to increase accuracy of existing provider-directed EHR CDS (vaccine alerts).

**Results:**

With our proposed sample size and using a factorial design, power calculations using baseline vaccination rate estimates indicated that 4286 participants per arm would give 80% power to detect a 3% improvement in influenza vaccination rates between groups (α=.05; 2-sided). Intention-to-treat unadjusted chi-square analyses will be performed to assess the impact of portal messages, either alone or in combination with the IVR call, on influenza vaccination rates. The project was funded in January 2014. Patient enrollment for the project described here completed in December 2014. Data analysis is currently under way and first results are expected to be submitted for publication in 2016.

**Conclusions:**

If successful, this study’s intervention may be adapted by other large health care organizations to increase vaccination rates among their eligible patients.

**ClinicalTrial:**

ClinicalTrials.gov NCT02266277; https://clinicaltrials.gov/ct2/show/NCT02266277 (Archived by WebCite at http://www.webcitation.org/6fbLviHLH).

## Introduction

Clinical decision support (CDS), including computerized reminders for providers and patients, can improve health outcomes by supporting the delivery of evidence-based and guideline-concordant medical care [1,2]. Many health systems effectively use provider-directed CDS. These provider-directed prompts frequently take the form of noninterruptive or interruptive “pop-up” alerts, reminding providers of recommended prevention or screening measures. While effective in many situations, provider-directed CDS is subject to important limitations. Instructions contained in alerts are often ignored or overridden due to alert fatigue [3-6]. Providers may also start to mistrust alerts if these are frequently triggered by erroneous or incomplete electronic health record (EHR) data. In light of these challenges, and in the setting of nationwide adoption of electronic patient portals, patient-directed CDS delivered via portal offers an innovative approach to improving patient care.

Electronic patient portals are secure websites that provide patients with 24-hour online access to limited EHR information. A portal provides patients with a personal health record “tethered” to their EHR [7]. Accessible information within a tethered portal varies by health system but may include vaccinations, laboratory results, and information from recent doctor visits or hospitalizations. Patients also use portals in numerous interactive ways including requesting prescription refills, scheduling nonurgent appointments, and seeking educational materials; portals can also provide a valuable link to Internet-based local and public health resources [8,9]. A core function of portals is secure messaging—electronic communication with the physician or health care team. A recent review found portals to have facilitated improved patient-provider communication with 10 of 27 articles reporting a positive association with portal’s secure messaging [10,11]. Advantages of tethered portals include enhancement of patient-provider communication, patient empowerment, support for care between visits, and improved patient outcomes [10]. Patient portals have been shown to improve medication adherence, decrease office visits, increase self-management of disease and disease awareness, increase use of preventative medicine, and increase inclusion of patients in medical decision making [12,13]. While studies of portal use show promising results, to date few randomized trials have tested the impact of patient-directed CDS via portal on receiving guideline-concordant care.

Vaccinations are a preventive measure well suited for incorporation into a patient-directed CDS intervention via patient portals. Previous patient outreach interventions have been shown to improve rates of vaccine completion and have been tested using multiple options including mailed letters, post cards, person-to-person phone calls, automated phone messages, and post card and phone combination [14,15]. Few studies have tested the use of patient-directed vaccine reminders sent via patient portals; those that have done so have focused exclusively on untethered (ie, personally controlled) patient health records [16]. Influenza vaccines are a logical target for patient-directed CDS because they are familiar to the general population and are recommended widely but completed at suboptimal rates.

Influenza infections contribute to increased health care costs and loss of productivity, and can lead to serious medical complications and even death [17]. The effect is felt most in high-risk groups such as adults aged 65 years or older and those diagnosed with cancer or diabetes [18]; however, low-risk groups also suffer the consequences. In 2007, it was estimated that influenza infections were responsible for 31.4 million outpatient visits, with reports indicating direct medical expenses amounting up to US $10.4 billion (including inpatient, outpatient and pharmaceutical claims) and lost earnings due to death and illness costing about US $16.3 billion [19,20]. According to CDC estimates, during the 2013-14 influenza season, influenza vaccination resulted in approximately 7.2 million fewer illnesses and 90,068 fewer hospitalizations. Despite numerous reasons to protect against influenza, only 45% of the US population received influenza vaccinations during the 2012-13 influenza season [21].

Our intervention, which will be implemented in a large multispecialty group practice in central Massachusetts, aims to improve rates of influenza vaccination among eligible adult patients by using a patient-directed CDS. We also aim to improve the accuracy of existing provider-directed CDS (influenza vaccine alerts) by capturing information on vaccines completed outside the clinic and using this self-reported patient information to update EHRs. We herein describe the protocol for a randomized controlled trial using EHR patient portal messages and interactive voice recognition (IVR) calls to (1) deliver messages promoting influenza vaccine completion and (2) solicit patient self-report on vaccines completed outside the clinic and on barriers to vaccination.

## Methods

### Study Objectives

Our overarching goal is to improve rates of influenza vaccination among eligible adults at Reliant Medical Group (RMG). We are conducting this randomized outreach intervention with the following main objectives that support this goal: (1) to determine whether our outreach increases likelihood of influenza vaccination (and if so, whether one mode of outreach is most effective); (2) to improve documentation of influenza vaccinations administered outside the practice by inviting patient self-report (improving accuracy of existing decision support tools); and (3) to deliver to patients targeted factual vaccine information related to that patient’s concerns.

### Study Outcomes

The primary study outcome is the percentage of eligible patients receiving influenza vaccines administered at our group practice during the 2014-15 influenza season. We will also study process measures including percentage of message recipients reached (ie, percentage of those who answer the IVR call and percentage of those who open the portal message) and number of intervention patients who self-report influenza vaccines administered in the community.

### Study Design

We will conduct a nonblinded, randomized controlled trial directed at patients who, by November 2014, do not have influenza vaccinations for the 2014-15 influenza season recorded in the EHR. Among these patients, those also overdue for pneumococcal vaccination(s) will receive outreach messages with additional language encouraging them to speak with their health care provider as well as a link to access more information about pneumococcal vaccination. Description of the pneumococcal vaccine intervention and analysis will not be a focus of this paper.

Authors designed and will implement the study, and will have full oversight and responsibility for data collection, analysis, and manuscript preparation.

### Theoretical Model

**Figure 1:**
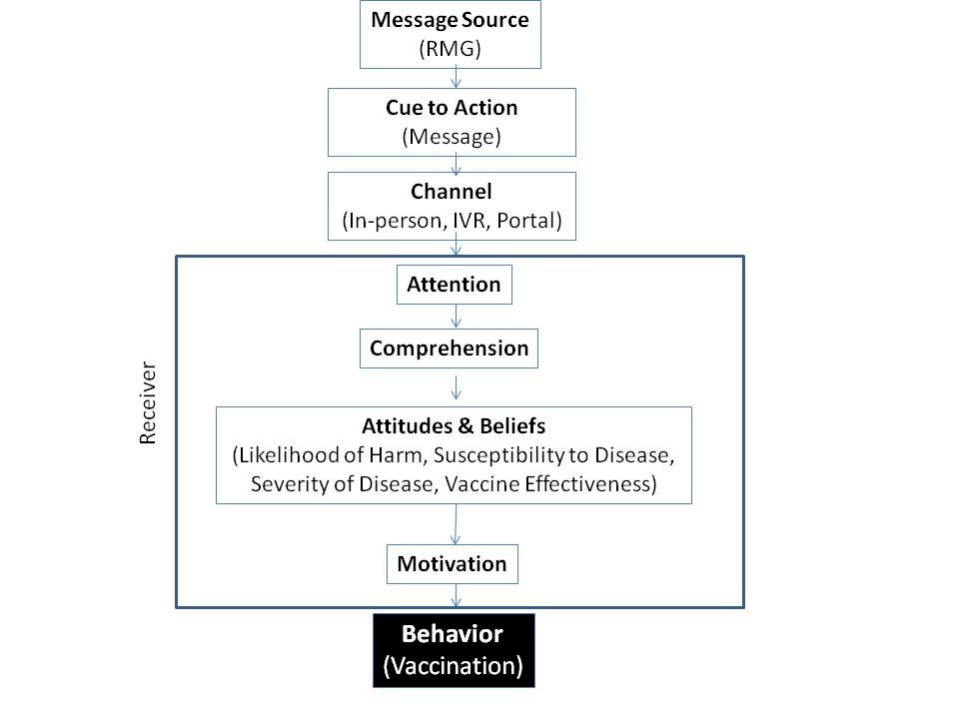
The Communication Human Information Processing model [22,23].

The Communication Human Information Processing model [22,23] (Figure 1) provides the overarching framework for the design and implementation of the intervention components. Growing out of extensive research on effective communication of safety information, this model includes the core concepts of communication theory (ie, message source, channel, and receiver) while highlighting the need to enhance effective information processing. Effective information processing requires attention to and comprehension of the message (eg, that a vaccine is needed); these processes are influenced by attitudes and beliefs, and motivation. In the context of vaccination, beliefs about susceptibility, severity, disease likelihood, and vaccine effectiveness are likely to be important [24]. All of these processes in turn influence behavior (ie, vaccination). Our interventions are designed to garner attention, be easily understood, address critical beliefs, and motivate vaccination.

### Study Procedures

This study consists of two phases, namely, (1) preintervention study components and (2) IVR and portal randomized outreach intervention.

#### Phase 1: Preintervention

To develop provider, staff, and patient outreach content, we conducted 30 in-depth interviews with patients, providers, and staff. Patient interviews elicited reasons for getting vaccinated, barriers to vaccination, and feedback to inform development of patient outreach materials used for the intervention. Provider and staff perceptions of patient barriers to vaccination helped to further shape patient outreach materials.

Information from interviews also informed our educational outreach; 10-minute in-person presentations were given by physician researchers who were also well-respected members of the group practice. Presentations were incorporated into routine clinical practice meetings attended by both physicians and staff. Based on results from provider and staff interviews, the in-person presentation provided an overview of this study and also provided information on pneumococcal vaccine guidelines. Follow-up emails sent bimonthly to all physicians in the group practice reviewed guidelines for both influenza and pneumococcal vaccines.

#### Phase 2: Interactive Voice Recognition and Portal Randomized Outreach Intervention

##### Clinical Setting

This study will be conducted at RMG, a large multispecialty group that employs 217 outpatient physicians at more than 13 clinical locations throughout Central Massachusetts. RMG providers care for approximately 140,000 adults aged 18 years and older. Approximately 113,000 of these adults receive their primary care through RMG via either the internal medicine/geriatric or family medicine departments. Approximately 89.5% of RMG patients are white, 4.3% are African American, and 4.6% Asian.

##### Electronic Health Record

All RMG providers and staff use an EHR developed by Epic Systems Corporation. Consistent with evidence showing that CDS can improve rates of indicated vaccines [25-27], the RMG EHR is configured to flag a patient’s record when they are due or overdue for an immunization based on the patient’s age, immunization history, medical, surgical, and social history. When patients call or are seen at RMG, physicians and staff accessing the patient’s record are alerted to immunizations that are due or overdue. Using a Microsoft SQL Server database, which is updated nightly with all of the clinical data from the EHR, it is possible to identify patients aged 18 years and older who are eligible and in need of immunizations.

##### Electronic Patient Portal

All RMG patients are given the option to sign up for MyChart, an electronic patient portal within Epic that is free of cost to patients and provides them with personalized and secure online access to portions of their medical record. Over 30% of RMG patients use MyChart. Patients can view their immunization history as well as alerts for immunizations that are due or overdue. They can securely send messages to and receive messages from RMG providers. The Epic MyChart system has the capacity to survey selected populations of patients.

##### Current Use of Interactive Voice Recognition Technology

RMG has an ongoing relationship with a company that uses IVR calls to alert patients about upcoming appointments and allows patients to respond to a limited number of scripted questions. Standard operating procedures and methods for transferring patient data existed prior to the intervention and helped guide our protocols.

### Participants

#### Eligibility Criteria

Patients are eligible for the study if they (1) have had an RMG primary care provider during the 1 year prior to randomization; (2) are aged 18 years or older on the date of randomization; (3) have had a recent office visit or telephone encounter with an internal medicine practitioner or family practitioner. The requirement for a recent office visit or telephone encounter was intended to minimize inclusion of patients who had moved to another practice but whose names were retained in RMG records. Our definition of “recent” depended on patient’s age. We defined “recent office visit” based on the age group of the patients; because older RMG patients (aged ≥ 65 years) visit their providers more frequently, we required an office visit or phone encounter within the 18 months prior to randomization for this population. For adults aged 18-64, we required an office visit or phone encounter within the 3 years prior to the intervention. To ensure capture of patients transitioning from pediatric to adult care, the visit or phone call could also be with a pediatrician.

#### Exclusion Criteria

Patients will be excluded if there is EHR documentation of an allergy to influenza vaccine, or if they were one of 20 patients who participated in preliminary qualitative interviews conducted to inform development of outreach materials. Exclusion criteria also included the presence of any of the following on the date of randomization: (1) EHR documentation of influenza vaccination completion in the 2014-15 influenza season (or documented influenza vaccination after the end of the 2013-14 influenza season but before the start of the 2014-15 season); (2) name listed on the do-not-call list or no listed phone number. A patient is eligible for inclusion in the electronic patient portal (referred to as “portal” herein) portion of the randomized controlled trial if he or she is an active user, which is defined as having an activated portal with a login at least once in the year preceding randomization.

### Randomization Approach

**Figure 2:**
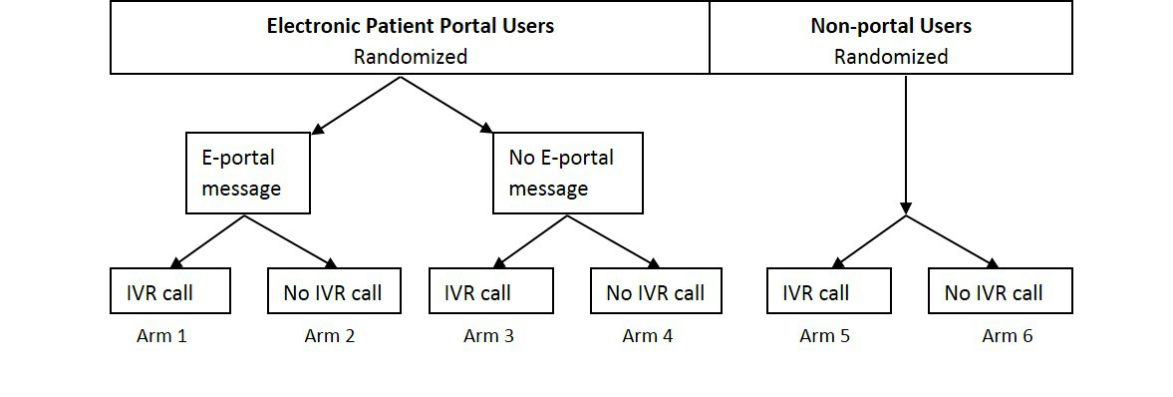
Outreach design.

Using computer-generated random number assignments, we will randomly select from the eligible population 20,000 portal users and 10,000 nonportal users (total of 30,000 patients). Using a factorial design (Figure 2), we will then use a computerized randomization method to assign 5000 patients to each of the 4 arms (portal users) and, separately, to each of the 2 arms (nonportal users). Thus, we will have a total of 6 arms, each with 5000 patients.

### Recruitment and Informed Consent

The study was reviewed and approved in 2014 by the RMG Institutional Review Board (IRB) and subsequently (in 2015) oversight was transferred to the University of Massachusetts IRB. A waiver for informed consent for patient outreach was approved by the IRB.

### Intervention

We designed the IVR calls and portal messages to include similar content (Textbox 1)

**IVR call and portal message content**

IVR calls and portal messages included...

- Personalized greeting
- Message seeking to establish influenza vaccines as social norm (“people your age get vaccinated against the flu...”) and informing patients what age group vaccines are recommended for
- Information intended to optimize access to vaccines (dates/locations for upcoming Reliant Medical Group (RMG) influenza clinic and list of additional ways to schedule a vaccine appointment)
- Information on accessing the CDC website for vaccine information

IVR calls and portal messages sought to elicit patient responses

- Patients were asked whether they had received influenza vaccines outside of RMG and were offered an opportunity to report date and location of influenza vaccinations received at external sites
- For patients who responded that they had not received any influenza shot for the 2014-15 season, several questions addressing vaccine barriers were presented
- Targeted educational information dispelling myths and misconceptions about the influenza vaccine based on patient responses to barrier questions

### Electronic Patient Portal Intervention

We designed an outgoing secure portal message to be sent via MyChart to patients randomized to the portal message arms (see Multimedia Appendix 1). Portal message content will appear in letter format with the signature line reflecting the name of the patient’s primary care provider. Portal messages will be delivered through standard channels used for portal-based correspondence between RMG health care providers and patients (ie, generic message that contains no personal health information nor any reference to vaccines is delivered to patient’s email account. Message prompts patients to log into secure portal account via hyperlink). Once logged into portal accounts, patients must click on a message labeled “Brief Flu Questionnaire” to view the outreach message.

Characteristics unique to the portal message compared with IVR messages include the ability to offer direct online scheduling of appointments for influenza vaccines. Information about accessing CDC vaccine website(s) appears within the body of the portal message as a hyperlink (and is conveyed verbally in the IVR script). Opportunities to report external influenza vaccinations, vaccine barrier questions, targeted information dispelling misconceptions, and branching logic matched the IVR call content.

#### Portal Message Delivery

To reach unvaccinated patients, we will intervene 2 months into the influenza season, a practice supported by prior research [28]. Messages will be sent out to 500-1500 patients daily over 9-10 days, beginning 1-week postrandomization.

### Interactive Voice Recognition Call Intervention

We designed a script to be delivered via IVR call to patients randomized to the IVR arms. Combining voice recognition with branching logic, calls will elicit patient self-report of influenza vaccinations completed outside of RMG. For patients reporting no influenza vaccine completion, IVR calls will deliver a series of questions on vaccine barriers, providing brief information intended to address the barriers which the patient identifies.

IVR calls will appear on telephone caller ID as “RELIANT MED.” This is consistent with current identification of IVR calls used for appointment reminders and is a detail that we consider critical to the success of call answering.

### Interactive Voice Recognition Call Delivery

Intervention IVR calls will be placed using standard procedures suggested by the IVR design team, which include identification of optimal times to call based on the patient’s age and suggestions for keeping patients engaged to achieve study goals. IVR calls will begin by confirming the patient’s identity. If voicemail is encountered or if the person reached identifies himself or herself as someone other than the patient, the IVR system will leave a message asking patients to call back and providing an inbound call line number. An inbound call line will be maintained throughout the duration of outgoing calls and for 2 weeks after the final outgoing call is placed, and patients calling this number from the phone number of record will hear the IVR call script in its entirety, beginning with questions confirming the identity of the caller.

### Data Upload Into Epic Electronic Health Record

Patient reports of prior immunization dates and location will be loaded through Epic’s Inbound Immunization interface and will be incorporated into the patient’s medical record. Data derived from portal questionnaires, including information on patient barriers, will be stored and available to the project team for analysis.

Patient-reported immunization type, mapped to “Codes for Vaccine Administered” (CVX code), date of vaccination, location of vaccination, and reasons for not being immunized will be sent to the study team by the company providing IVR calls. The patient’s immunization history will be updated via Epic’s Inbound Immunization interface, and transcriptions of all IVR responses will be stored for analysis by the project team.

### Measures

We will draw on data from EHR records, brought into the EHR via several pathways. Information on influenza vaccines will be gathered by capturing data entered into the EHR via two routes: (1) direct documentation by RMG staff of influenza vaccines administered at RMG or reported in-person by the patient and entered manually into Epic EHR by staff or provider; (2) patient self-report of vaccines administered outside of RMG (self-report received via MyChart questionnaire or IVR).

#### Primary Outcome

##### Influenza Vaccine Outcomes

Our primary outcome will be percentage of eligible patients with influenza vaccines directly administered and documented in an RMG facility as of the end of the 2014-15 influenza season. Because the control groups will not be given the opportunity to self-report, immunizations captured solely through the MyChart questionnaire or the IVR will be excluded from the primary analysis. Our exclusion of self-reported vaccines from our outcome analysis avoids introducing bias through differential capture (intervention vs control) of self-reported outcomes.

#### Secondary Outcome

##### Influenza Vaccine Outcomes

We will perform a descriptive analysis, calculating the percentage of patients for whom self-report through our intervention was the sole method for documentation of influenza vaccination completion. We will assess this at the end of the 2014-15 influenza season. This analysis is intended to provide preliminary insights into the percentage of patients who are vaccinated outside of the RMG clinics and who choose to self-report these vaccines.

#### Process Measures

We will examine patients who receive portal messages and calculate (1) percentage of recipients who open messages and (2) percentage of recipients who complete questionnaires. We will examine patients who receive IVR calls and calculate (1) percentage of recipients who answer the call and (2) percentage of recipients who complete the calls by responding to questions.

### Proposed Analyses

To determine the impact of our interventions on RMG vaccination rates for the 2014-15 influenza season, we will perform unadjusted and adjusted analyses of randomized patients (30,000 patients). Because of differential rates of vaccination at baseline between portal users and nonusers, analyses in these groups will be conducted separately. Intention-to-treat unadjusted chi-square analyses will include the analyses presented in Table 1.

We will examine the adequacy of randomization in the overall group and separately among e-portal users and non e-portal users by assessing whether there was differential representation in the intervention versus control groups by 5 patient characteristics readily available in the EHR. These will include (1) age group; (2) race/ethnicity; (3) sex; (4) influenza vaccination in previous year; and (5) completion of an office visit in the previous year; if differences are found we will carry out adjusted analyses using logistic regression to control for significant differences, modeling odds of receiving influenza vaccine.

Multivariate logistic regression analyses will also be performed. As in our unadjusted analyses, due to differential rates of vaccination at baseline between portal users and nonusers, adjusted analyses in these groups will be conducted separately. We will create dummy variables for assignment to the portal message arm (among portal users) and for assignment to the IVR call arm (among both portal users and, separately, among nonportal users). Including these dummy variables and adjusting for demographic and practice-level covariates, we will model odds of receiving an influenza vaccine in the 2014-15 influenza season. We will also examine the heterogeneity of treatment effects within each subgroup using logistic analysis.

### Power and Sample Size

With our proposed sample size and using a factorial design, power calculations using baseline vaccination rate estimates indicated that 4286 participants per arm would give 80% power to detect a 3% improvement in influenza vaccination rates between groups (α=.05; 2-sided). Based on previous studies [29,30], we expect a 10-20% improvement in vaccination rates for either intervention compared with control.

**Table 1:** Analyses by arm.

Study question	Comparison
Among portal users, did portal message receipt alone increase the likelihood of influenza vaccine completion compared with control?	Compare percentage of vaccine completion among those randomized to receipt of portal messages versus those randomized to neither portal messages nor IVR calls (usual care). (Arm 2 vs 4)
Among portal users, did portal message receipt plus IVR call increase the likelihood of influenza vaccine completion compared with portal message alone?	Compare percentage of vaccine completion among those randomized to receipt of both portal messages and IVR calls versus those randomized to only portal messages. (Arm 1 vs 2)
Among portal users, did IVR call alone increase the likelihood of influenza vaccine completion compared with control?	Compare percentage of vaccine completion among those randomized to receipt of IVR calls versus those randomized to neither portal messages nor IVR calls (usual care). (Arm 3 vs 4)
Among those who do not use portals, did IVR call alone increase the likelihood of influenza vaccine completion compared with control?	Compare percentage of vaccine completion among those randomized to receipt of IVR calls versus those randomized to neither portal messages nor IVR calls (usual care). (Arm 5 vs 6)

## Results

Intention-to-treat unadjusted chi-square analyses will be performed to assess the impact of portal messages, either alone or in combination with the IVR call, on influenza vaccination rates.

## Discussion

This study will test the effectiveness of a patient-directed CDS intervention, aimed at improving rates of influenza vaccination within a primary care adult population. The approach described has important implications for future patient-directed CDS initiatives seeking to use tethered patient portals. As a low-cost and rapid means of communicating with patients who have activated their electronic accounts, patient portals are a promising channel through which CDS may be delivered. Targeting patients through portals aligns well with the “CDS Five Rights,” which stipulate that effective CDS delivers the right information to the right people, via the right channels, in the right intervention formats and at the right points of workflow [31]. Our approach studies the possibility that the patient is the person best positioned to receive and act on information related to influenza vaccination.

Influenza vaccination has several characteristics that made it ideally suited for this study design. Influenza vaccinations are a single-dose annual vaccine, for which standing orders were already in place in our medical group. They are recommended almost universally across age groups, and therefore, we did not require that physicians review the list of patients to whom promotional messages were directed prior to sending, thereby reducing physician burden. There is widespread familiarity with influenza vaccination among the lay public, and numerous community sites and workplaces offer these vaccinations, thus they are commonly administered outside of the medical group. Seeking to collect data on vaccinations given in the community was therefore a reasonable component of our outreach. This factor might be less appropriate for other preventive health behaviors. Guidelines calling for annual vaccination create a short recall period (2-3 months in our study design) for individuals reporting vaccination in a given season. The short recall period and widespread familiarity was our rationale for accepting patient self-report. With inputs from the physicians on our research team, we further reasoned that providers would be willing to update the EHR based on patient self-report of influenza completion during an in-person visit (without accompanying printed documentation) and that our collection of self-reported data via outreach was an acceptable alternative to in-person patient report. Our outreach may have the additional benefit of identifying patients with influenza vaccine allergy that has not previously been documented.

This protocol has some limitations. Although we collected self-report of influenza vaccinations completed in the community, our primary analysis focuses on RMG-documented vaccines, thus avoiding the bias that would be introduced through differential capture (intervention versus control) of self-reported data.

For our secondary outcome measurement, we will report percentage of patients self-reporting influenza vaccines completed in the community. Although self-report introduces the possibility for inaccuracies, self-reported vaccination status is a measure commonly used to assess vaccination status and is the standard used by the Behavioral Risk Surveillance System and the National Health Interview Survey [32-34]. Previous validation efforts for self-reported influenza vaccines documented high sensitivity (0.98 to 1.0) and moderate specificity (0.71-0.79) [32]. In addition to reviewing commonly accepted measures and published validation reports, we also considered the levels of ascertainment consistent with routine clinical care. Many clinicians accept a patient’s verbal self-report of completed influenza vaccination and update the EHR accordingly (without requiring paper documentation). For all of these reasons, we chose to allow patient self-reported influenza vaccinations to be entered into the EHR.

An additional limitation of our study stems from the timing of outreach. After eligibility determination but prior to disseminating the outreach, we will have a 1-week gap built in for quality checks and data transfers to the company handling IVR calls. It is possible that during this 1-week gap, patients randomized to receipt of the intervention will obtain their influenza vaccines. Patients with vaccinations completed during this period should be evenly distributed across all arms, minimizing any associated bias. Once the intervention starts, IVR calls and patient portal messages will be disseminated in batches. While this is a standard protocol for delivering IVR appointment reminders and prevents outgoing portal messages from being labeled as spam by email servers, it also introduces the possibility that additional patients may get vaccinated prior to receiving the intervention. Again, patients vaccinated prior to receipt of messages should be evenly distributed across all arms.

While our single-site study is limited insofar as it cannot be considered representative on a national scale, a future study could address this by sampling from our population in proportion to national demographics. In addition, we have designed our intervention to be easily tested across diverse settings. This intervention will be implemented using Epic, one of the top EHR vendors in the country [35]. If successful, this study’s intervention may be adapted by other large health care organizations using Epic and tested as a means of increasing vaccination rates among diverse populations.

## Appendix

Multimedia Appendix 1 Portal message example.

## Abbreviations

CDS: clinical decision support
EHR: electronic health record
IRB: Institutional Review Board
IVR: interactive voice recognition
RMG: Reliant Medical Group
