# Supplementary material for: Improving Rates of Influenza Vaccination Through Electronic Health Record Portal Messages, Interactive Voice Recognition Calls and Patient-Enabled Electronic Health Record Updates: Protocol for a Randomized Controlled Trial
Source: JMIR Res Protoc. 2016 May 6;5(2):e56. doi: 10.2196/resprot.5478 (PMC4875493; doi:10.2196/resprot.5478)
Supplement: Multimedia Appendix 1 [file resprot_v5i2e56_app1.pdf]

Multimedia Appendix 1. Portal message example.

Dear Phil
Winter's almost here. I'd like to remind you that people your age get vaccinated against the flu each year. To help protect you and your family, we recommend the flu vaccine for everyone aged 6 months and older.
Complete the attached questionniare to tell us if you have already been vaccinated, or to help us understand why you have not done so. We can update your record or try to answer your flu vaccine questions.

If you still need a flu vaccine, you can:

Stop by our Flu Vaccine Clinic on December 6th, 8am - 12pm at Plantation Street (630 Plantation Street, Worcester MA 01605) no appointment necessary.

OR

Schedule an appointment for a flu vaccine by calling our office (Reliant Medical Group main phone number 508-852-0600).

OR

Use Mychart to request an appointment. If you want, you can use your mobile device to do this. After you request an appointment, we will call you back to confirm the date and time.

OR

Ask us for your flu vaccine at your next doctor's appointment.

To learn more about the flu vaccine, click here: http://www.cdc.gov/flu/

Please scroll to the top of this message to complete the questionnaire.

What happens to this information? We periodically evaluate our programs for effectiveness and are trying to learn how to better serve our patient's needs for preventive care. This questionnaire is voluntary and your responses will be entered into your Reliant medical record. If you have received the flu vaccine this year, responding to the questionnaire will help keep your records up to date. If you have not received the vaccine, you will be invited to share your reasons and concerns and we will provide some additional information to try to answer your questions.

Sincerely,
